# A role for the lateral parabrachial nucleus in cardiovascular function and fluid homeostasis

**DOI:** 10.3389/fphys.2014.00436

**Published:** 2014-11-18

**Authors:** Pamela J. Davern

**Affiliations:** Neuropharmacology Laboratory, Baker IDI Heart and Diabetes InstituteMelbourne, VIC, Australia

**Keywords:** lateral parabrachial nucleus, serotonin, thirst, sodium appetite, cardiovascular responses, blood pressure

## Abstract

The lateral parabrachial nucleus (LPBN) is located in an anatomical position that enables it to perform a critical role in relaying signals related to the regulation of fluid and electrolyte intake and cardiovascular function from the brainstem to the forebrain. Early neuroanatomical studies have described the topographic organization of blood pressure sensitive neurons and functional studies have demonstrated a major role for the LPBN in regulating cardiovascular function, including blood pressure, in response to hemorrhages, and hypovolemia. In addition, inactivation of the LPBN induces overdrinking of water in response to a range of dipsogenic treatments primarily, but not exclusively, those associated with endogenous centrally acting angiotensin II. Moreover, treatments that typically cause water intake stimulate salt intake under some circumstances particularly when serotonin receptors in the LPBN are blocked. This review explores the expanding body of evidence that underlies the complex neural network within the LPBN influencing salt appetite, thirst and the regulation of blood pressure. Importantly understanding the interactions among neurons in the LPBN that affect fluid balance and cardiovascular control may be critical to unraveling the mechanisms responsible for hypertension.

## Introduction

The lateral parabrachial nucleus (LPBN) has become increasingly recognized as a major relay site to receive information related to the control of blood pressure (BP), thirst and or sodium appetite from the nucleus of the solitary tract (Spyer, 1981). Consistent with its anatomical connectivity, the LPBN plays a crucial role in inhibiting water and sodium intake in response to increases in BP and volume and conversely, stimulating water intake in response to hypovolemia and hypotension.

## LPBN neuroanatomy and projections

**Figure 1:**
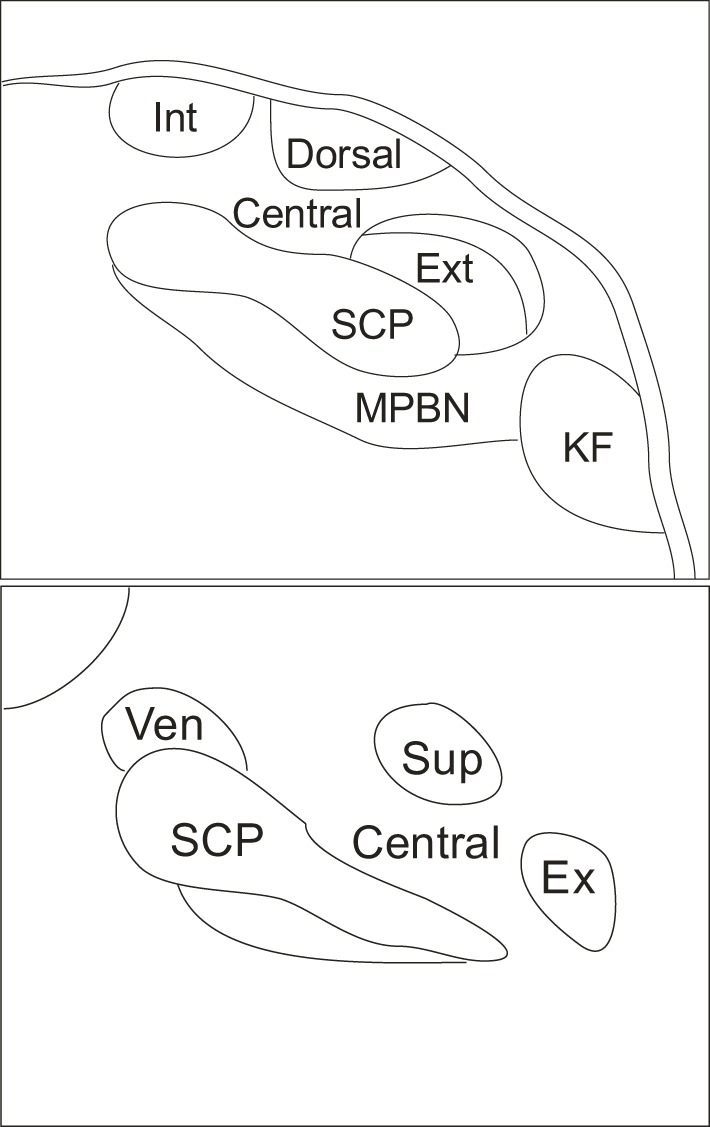
**Schematic indicating coronal organization of the parabrachial nucleus surrounding the superior cerebellar peduncle. Top panel** illustrates caudal parabrachial nucleus and **lower panel** illustrates rostral parabrachial nucleus. Abbreviations: central, central lateral subnucleus; dorsal, dorsal lateral subnucleus; ex, extreme lateral subnucleus; ext, external lateral subnucleus; int, internal lateral subnucleus; KF, kolliker fuse nucleus; MPBN, medial parabrachial nucleus; SCP, superior cerebellar peduncle; sup, superior lateral subnucleus; ven, ventral lateral subnucleus.

The parabrachial nucleus (PBN) is a brain structure located in the dorsolateral pons that surrounds the superior cerebellar peduncle (Figure 1). The superior cerebellar peduncle defines the three major subdivisions of the PBN: the LPBN, the medial parabrachial nucleus (MPBN) and the ventrolateral Kolliker-Fuse nucleus (Fulwiler and Saper, 1984). The subnuclear organization of the PBN is comprised of ten separate and distinct subnuclei. The LPBN consists of seven subnuclei including: the internal lateral, superior lateral, extreme lateral, external lateral, central lateral, ventral lateral, and the dorsal lateral subnuclei. The remaining three subnuclei include the MPBN, the external medial nucleus, and the Kolliker-Fuse nucleus (Fulwiler and Saper, 1984).

Several anatomical studies (Herbert et al., 1990; Moga et al., 1990) have reported reciprocal connections between the LPBN and many of the regions in the medulla and forebrain that respond to changes in body fluid homeostasis thereby supporting a similar role for the LPBN. Specifically, connections from the LPBN may relay signals that ascend from the area postrema and nucleus of the solitary tract to forebrain regions that control fluid and electrolyte balance. The nucleus of the solitary tract is the primary afferent terminal site from visceral receptors, including arterial baroreceptors located within the aortic arch, and the carotid sinus (Spyer, 1981; Rettig et al., 1986; Paton, 1998; Deuchars et al., 2000) that detect changes in BP. This information is conveyed via the IXth and Xth cranial nerves to the brain to regulate and maintain BP through autonomic and neuroendocrine responses (Paton, 1998). The LPBN is increasingly viewed as a critical integrative site in the transfer of visceral cardiovascular information from the brainstem to a wide variety of regions in the forebrain (Fulwiler and Saper, 1984; Herbert et al., 1990; Moga et al., 1990; Saper, 1995).

## Subnuclei mediating cardiovascular function

Early studies experimentally induced hypertension or hypotension and utilized Fos expression as a marker of neuronal activity to determine whether neurons in the PBN were differentially activated. Elevated levels of Fos immunoreactive neurons were observed in the central and dorsal subnuclei of the LPBN, as well as in the dorsal part of the external in response to either an increase or decrease in arterial pressure (Rocha and Herbert, 1996). Less activation was observed in the superior LPB subnucleus and there was no evidence of neuronal activity in the internal or ventral LPB subnuclei. Overall, neuronal activity was described as located within the mid, and caudal aspects of the central and external LPB subnuclei and at the caudal aspect of the LPBN, labeling was exclusively identified in the external LPB subnucleus. Furthermore, similar patterns were described in response to blood volume expansion.

Functional studies indicate a significant role for the LPBN in regulating cardiovascular activity. Electrical and chemical stimulation of the LPBN evoked a significant increase in mean arterial pressure (MAP), tachycardia and sympathetic nerve activity (Chamberlin and Saper, 1992). The increase in MAP was elicited via stimulation of the dorsal, central and the external LPB subnuclei. By contrast, topographical mapping of the pressor, and tachycardia responses in the LPBN, via electrical or glutamate stimulation identified the most sensitive site in the outer edge of the external LPB subnucleus (Chamberlin and Saper, 1992), whereas bradycardia was reported as arising exclusively from the dorsal LPB subnucleus. Furthermore, stimulation of the LPBN modulated baroreflex regulation of sympathetic nerve activity by attenuating baroreflex inhibition of MAP (Hayward and Felder, 1998). Additionally, LPBN neurons were activated in response to increased levels of circulating angiotensin II (AngII) (Herbert, 1996), or changes in baroreceptor input (Hayward and Felder, 1995). Moreover, temporary chemical inactivation of the LPBN induced a pressor response (Hayward and Felder, 1998). Therefore, it is possible that neurons in the LPBN are part of a central pathway that inhibits medullary baroreflex pathways. Furthermore, pharmacological blockade of *N*-methyl-D-aspartic acid receptors in the spinal cord reduced MAP and heart rate (HR) induced by electrical stimulation of the LPBN (Bazil and Gordon, 1990). These data suggest that maintenance of sympathetic vasomotor tone mediated by the LPBN may be dependent upon synaptic activation of *N*-methyl-D-aspartic acid receptors located on sympathetic preganglionic neurons and or spinal interneurons.

Secretion of vasopressin from the posterior pituitary is an important compensatory response to changes in cardiovascular parameters. Electrical stimulation of the superior and external LPB subnuclei increased MAP and produced a small but significant increase in plasma vasopressin (Sved, 1986). This may be an effect of baroreceptor information received by neurons in the LPBN that, in turn, innervate vasopressin secreting neurons in the magnocellular portion of the paraventricular nucleus of the hypothalamus (Dampney et al., 1995). Furthermore, ganglionic blockade increased MAP following an increase in plasma vasopressin levels via stimulation of the LPBN (Sved, 1986). These data demonstrate that the LPBN is likely to contribute to the modulation of vasopressin release in response to cardiovascular challenges such as hypotension.

Sustained hypertension during intravenous infusion of AngII was prevented by ablation of the LPBN suggesting interference of neurogenic pressor mechanisms associated with increased levels of AngII (Fink et al., 1991). Even so, reversible bilateral lesions of the LPBN by injections of lidocaine did not change the pressor response to centrally injected AngII (Menani et al., 1995; Menani and Johnson, 1995). However, bilateral LPBN injection of lidocaine alone, induced a pressor response that peaked at 3 min post injection (Menani et al., 1995), whereas at 15 min, no significant change to either MAP or HR was observed compared with saline (Saleh and Connell, 1997). Thus, the LPBN may tonically inhibit MAP. Reversible blockade of the LPBN elicited significant increases in pharmacologically induced changes in HR independent of variations in BP. Electrical or chemical stimulation of the LPBN attenuated reflex bradycardia with no effect on pharmacologically-induced pressor responses. Therefore, the LPBN may play a role during cardiac baroreflex activation under normal conditions by inhibiting both sympathetic and parasympathetic tone. This was supported by pharmacologically-induced enhancement of reflex tachycardia following reversible blockade of the LPBN and increased plasma noradrenaline levels following lesions of the LPBN (Hubbard et al., 1987).

As described above, serotonin in the LPBN inhibits AngII-induced water intake. In contrast, bilateral injections of the serotonergic receptor anatagonist, methysergide into the LPBN did not change pressor responses induced by central administration of AngII. However, LPBN injections of a serotonergic receptor antagonist alone increased MAP (Menani and Johnson, 1995). Thus, serotonergic neurons, or fibers of passage in the LPBN may tonically inhibit MAP.

Increased vagal activation enhanced the release of L-glutamate, a primary neurotransmitter that relays cardiovascular information through the LPBN (Saleh and Connell, 1997). Stimulation of cardiac sympathetic afferents activated glutamatergic neurons in the external LPB subnucleus (Guo et al., 2005), likely due to visceral activation affecting cardiac baroreflex function (Saleh and Connell, 1997). Furthermore, the plasma noradrenaline levels during vagal activation were markedly increased immediately following withdrawal of vagus stimulus, indicating an immediate increase in sympathetic activity (Saleh and Connell, 1998). Ablation of the LPBN also blocked the enhanced release of plasma noradrenaline and reduced baroreflex sensitivity following stimulation of the vagus nerve. Furthermore, LPBN ablation increased plasma noradrenaline concentration and basal plasma renin activity without affecting either MAP or HR (Hubbard et al., 1987).

Fos expression indicated that vagal stimulation activates neurons in the central and external LPB subnuclei (Saleh and Cechetto, 1996). Also in the same regions of the LPBN, the level of immunohistochemical staining for substance P, calcitonin gene-related peptide, neurotensin, somatostatin, and cholecystokinin were dependent on the level of vagus nerve activity (Saleh and Cechetto, 1996). Evidence also suggests interactions among these neuropeptides that modulate visceral inputs from the nucleus of the solitary tract to the thalamus via the LPBN (Saleh and Cechetto, 1995).

A majority of neurons in the LPBN were identified as non-responsive to right atrial stretch (Jhamandas et al., 1991). However, extracellular recordings in the LPBN following either stimulation of arterial baroreceptors or systemic administration of AngII showed increased excitability was recorded in 17.5% and decreased excitability in 48.1% of neurons. The response to systemic administration of AngII in some neurons could not be explained by increased BP. Therefore, AngII may act on neurons located in circumventricular organs, such as the area postrema, that project to the LPBN. In separate experiments, when the LPBN, or the area postrema was ablated, hypertension induced by systemic AngII infusion was prevented (Fink et al., 1991). Therefore, the interruption of the projection from the area postrema to the LPBN may prevent pressor activity caused by blood-borne AngII. This result indicates that the LPBN may play a critical role in the central augmentation of neurogenic pressor activity.

## Influence on hemorrhage and hypovolemia

Experimentally induced hemorrhage initiated neuronal activation, as indicated by Fos expression, and increased cellular activity in many brain regions that regulate cardiovascular activity including the LPBN. However, there has been a discrepancy in relation to neuronal activation within specific LPB subnuclear groups. This variability was likely the result of dissimilar methods used to induce hemorrhage. Furthermore, paradigms utilized to measure volume receptor information can lead to changes in BP. Utilizing a methodology that increased sympathetic activity, vasoconstriction and pituitary-adrenocortical hormone release, increased activity in one-third of neurons recorded within the PBN (Ward, 1988). Neurons responsive to hemorrhage were described as located within the external and central LPB subnuclei.

Experimentally induced hemorrhage via blood withdrawal previously determined as ineffective in activating arterial baroreceptors was used to confine results to volume receptor responses (Rocha and Herbert, 1996). Neuronal activity under these conditions was identified within the dorsal part of the external, central and dorsal LPB subnuclei and sparser labeling was evident within the superior LPB subnucleus. Also the number and the distribution of activated neurons increased progressively following hypovolemic hypotensive hemorrhage (Chan and Sawchenko, 1994). Although the results discussed only neuronal activity within the external LPB subnucleus, brightfield photomicrographs of Fos expression revealed sparsely activated neurons in the central and dorsal LPB subnuclei. The LPBN was also identified as less important in maintaining initial changes in arterial pressure during hemorrhage (Blair et al., 2001). However, the LPBN is an essential regulator of the bradycardia that typically accompanies haemorrhagic hypotension following initial blood loss. Chemical inactivation of the LPBN indicated that restoration of arterial pressure following hemorrhage involves neurons in the dorsal part of the external, and or in the ventral LPB subnuclei. Whereas, lesions of the LPBN impaired BP recovery after hypotensive blood loss and the recovery may be mediated by glutamate receptors located in the LPBN driving sympathetic vasomotor tone (Blair and Mickelsen, 2006).

Consistent with the above, volume depletion induced by administration of polyethylene glycol also increased neuronal activation in the LPBN (Curtis et al., 2002). Several lines of evidence indicate that activation of hindbrain regions such as input from volume receptors innervating the nucleus of the solitary tract, and or hormones acting at the area postrema, were important in responses to volume depletion. Hypotension, hemorrhage, and hypovolemia were seen to activate neurons in many of the same medullary and pontine brain regions. Additionally, unlike hypotension, and hemorrhage, sustained hypovolemia induced by administration of polyethylene glycol may primarily activate non-catecholaminergic neurons (Curtis et al., 2002).

## The baroreflex pathway

Several lines of evidence indicate that the LPBN may influence neurons located in the nucleus of the solitary tract to regulate cardiovascular activity. Electrolytic lesions of the LPBN enhanced (Hubbard et al., 1987; Saleh and Connell, 1997), and activation of the LPBN region inhibited (Chamberlin and Saper, 1994; Hayward and Felder, 1998) baroreflex-mediated cardiovascular responses. Others described neurons in the LPBN as exerting potent postsynaptic influences of an excitatory or inhibitory nature on both the spontaneous discharge of neurons in the nucleus of the solitary tract and on afferent inputs to the nucleus of the solitary tract from the carotid sinus (Felder and Mifflin, 1988). Furthermore, neurons located within the central LPB subnucleus were likely associated with dorsal periaqueductal gray activation that may modulate sympathetic drive (Hayward and Castellanos, 2003). As the LPBN is a secondary site for cardiovascular inputs to the brain, it is likely that the LPBN modulates this information either by direct local feedback or by relaying signals from more rostral brain regions.

The LPBN may also modulate sympathetic nerve activity that influences coronary vasoconstriction. Preliminary studies indicate that a descending projection from the hypothalamus to neurons in the LPBN may be responsible for coronary vasoconstriction (Miller et al., 1991). The LPBN has been identified as an essential component of the baroreflex pathway mediating coronary constriction (Gutterman and Goodson, 1996) and increased coronary vascular resistance was shown to involve activation of α-1 adrenoceptors (Miller et al., 1991).

## Implications for hypertension

The LPBN is likely to play a significant role in central mechanisms that control hypertension. As discussed above, microinjections of L-glutamate into the LPBN increased MAP (Ward, 1988), and activation of these LPBN neurons also activated cholinergic neurons in the rostral ventrolateral medulla and increased BP via muscarinic receptors (Kubo et al., 1998). Cholinergic neurons have been implicated in regulating BP and several studies have revealed that the release of acetylcholine in the rostral ventrolateral medulla contributes to hypertension (Kubo, 1998). Furthermore, it has been suggested that hypertensive rats with enhanced cholinergic activity in the rostral ventrolateral medulla, receive cholinergic inputs from LPBN pressor sites either directly, or indirectly perhaps maintaining hypertension (Kubo et al., 1998). However, neuronal activation in the LPBN, marked by Fos expression, in response to increased arterial pressure, and neurons retrogradely labeled from the rostral ventrolateral medulla in rabbits were largely identified as separate populations (Polson et al., 1995). Additionally, very few neurons in the LPBN activated by sustained hypotension project to the rostral ventrolateral medulla in rabbits (Horiuchi et al., 1999).

## Influence on water intake

A series of experiments by Johnson and colleagues provided physiological or behavioral data examining the role of the LPBN in response to various thirst challenges. Inactivation of the LPBN was accomplished via either a permanent lesion or temporary and reversible inactivation. Earlier studies utilized bilateral electrolytic lesions that resulted in complete ablation of the LPBN; others utilized injections of neurotoxin that selectively destroy cell bodies while sparing fibers of passage (Ohman and Johnson, 1986, 1989; Edwards and Johnson, 1991), or local anesthetic injected bilaterally into the LPBN to produce temporary, and reversible neuronal inactivation (Menani et al., 1995). Regardless of the method of LPBN inactivation, rats showed overdrinking in response to a number of dipsogenic treatments (see Table 1 for details). These included peripheral and central administration of AngII and subcutaneous (SC) isoproterenol (Ohman and Johnson, 1986, 1989; Edwards and Johnson, 1991; Menani and Johnson, 1995; Menani et al., 1995, 1996; Davern and McKinley, 2013). By contrast, water intake did not differ between rats with bilateral LPBN lesions, and control rats with intact LPB subnuclei following 24 h water deprivation, or following administration of isotonic or hypertonic saline, polyethylene glycol, or the cholinergic receptor agonist, carbachol (Ohman and Johnson, 1986, 1989; Edwards and Johnson, 1991; Menani et al., 1995). These data suggest that neurons within the LPBN are associated with regulating fluid intake through inhibitory mechanisms. In particular, lesions modified drinking in response to dipsogenic challenges that were associated with activation of the renin angiotensin system.

**Table 1:** **Table Summary of studies examining actions in the LPBN combined with treatments suggesting serotonin receptors in the LPBN are involved in regulating water and salt intake**.

**WATER INTAKE**			
**Bilateral LPBN**	**Treatment**	**Treatments that reduce responses**	**References**
Electrolytic lesion	SC Angiotensin II		Ohman and Johnson, 1986, 1989; Davern and McKinley, 2013
Electrolytic lesion	ICV Angiotensin II		Ohman and Johnson, 1989
Electrolytic lesion	SC Isoproterenol		Ohman and Johnson, 1986
Ibotenic acid injection	SC Angiotensin II		Edwards and Johnson, 1991
Ibotenic acid injection	SC Isoproterenol		Edwards and Johnson, 1991
Lidocaine injection	ICV Angiotensin II		Menani et al., 1995
Methysergide injection	ICV Angiotensin II	Bilateral LPBN injection of Serotonin	Menani et al., 1995
Methysergide injection	ICV Angiotensin II	Bilateral LPBN injection of Dimetoxy-4-iodoamphetamine hydrochloride	Menani et al., 1995, 1996
Methysergide	24 h sodium depletion		Menani et al., 1998b
Methysergide	24 h water deprivation		Menani et al., 1998b
**NaCl INTAKE**			
**Bilateral LPBN injection**	**Treatment**	**Treatments that reduce responses**	**References**
Methysergide	ICV Angiotensin II		Menani et al., 1996
Methysergide	Angiotensin II injected into SFO	Losartan injection into the SFO	Colombari et al., 1996
Methysergide	SC Furosemide + Captopril	Bilateral LPBN injection of dimetoxy-4-iodoamphetamine hydrochloride	Menani et al., 1996
Methysergide	SC Furosemide + Captopril	Losartan injection into the SFO	Menani and Johnson, 1998
Methysergide	24 h sodium depletion		Menani et al., 1998b
Methysergide	24 h water deprivation		Menani et al., 1998b
Methysergide	Intragastric load of hypertonic solution		De Luca et al., 2003
Methysergide	SC Isoproterenol		Menani et al., 2000; Davern and McKinley, 2010
Methysergide	SC Furosemide		Menani et al., 2000
Methysergide	ICV Carbachol		Menani et al., 2002
Methysergide	ICV Relaxin	ICV losartan	Menani et al., 2004
Methysergide	SC Deoxycorticosterone	Bilateral LPBN injection of 2, 5-dimethoxy-4-iodoamphetamine hydrochloride	De Gobbi et al., 2000

## Contribution to sodium appetite

A pronounced serotonergic pathway from the area postrema and nucleus of the solitary tract innervating the LPBN has been described (Lanca and van der Kooy, 1985). Neuroanatomical evidence and early investigations prompted the hypothesis that serotonergic mechanisms in the LPBN are likely to play an important inhibitory role in the control of sodium appetite. Blockade of the serotonergic receptors in the LPBN via bilateral injections of the non-selective 5-hydroxytryptamine (5-HT)_1/2_-receptor antagonist, methysergide resulted in a significant increase in sodium appetite in a number of experiments. Rats drank large volumes of hypertonic saline solutions irrespective of either dipsogenic or natriorexigenic treatment (see Table 1 for details). Sodium appetite was markedly increased when rats treated with bilateral injections of methysergide into the LPBN were administered AngII either intracerebroventrically (ICV), or directly into the subfornical organ (Menani and Johnson, 1995; Colombari et al., 1996). Other stimuli that resulted in the potentiation of intake of hypertonic saline included systemic administration of the diuretic, furosemide in combination with the Ang-converting enzyme inhibitor, captopril which was prevented by losartan pretreatment directly into the subfornical organ; 24 h sodium depletion (induced by the diuretic, furosemide, and exposure to 24 h of a sodium deficient diet); 24 h water deprivation; intragastric load of hypertonic solution and SC isoproterenol (Menani et al., 1996, 1998a,b, 2000; De Luca et al., 2003; De Gobbi et al., 2005; Andrade-Franze et al., 2010; Davern and McKinley, 2010). Conversely, bilateral injections of serotonin receptor agonist significantly reduced consumption of hypertonic saline solution when combined with systemic injections of furosemide and captopril (Menani et al., 1996). These data suggest that a serotonergic pathway in the LPBN plays an important inhibitory role in the control of sodium appetite. To this end, increased intake of hypertonic saline solution, and water stimulated by SC furosemide in combination with captopril was demonstrated as acting via serotonergic 5-HT_1A_ receptors in the LPBN (De Gobbi et al., 2005).

A number of experiments have focused on the effect of treatments typically recognized as eliciting dipsogenic responses. However, when inhibitory serotonin receptors in the LPBN are blocked, rats ingest a significant volume of hypertonic saline (see Table 1 for details). Such treatments include: systemic administration of both isoproterenol and furosemide and central administration of either AngII, carbachol, or relaxin (Colombari et al., 1996; Menani et al., 2000, 2002, 2004). When combined with bilateral injections of methysergide into the LPBN, injection of relaxin in normohydrated rats resulted in a significant increase in hypertonic saline solution with only a slight increase in water intake. The effects of this treatment were abolished when ICV losartan blocked central AT_1_ receptors (Menani et al., 2004). Therefore, it is likely that the natrioexigenic effect of relaxin is mediated by blocking serotonin receptors in the LPBN and the activation of brain AngII. However, it should be noted that stimulation of hypertonic saline solution intake is not dependent on AngII, as mineralocorticoid dependent ingestion of hypertonic saline can also be produced by SC deoxycorticosterone (De Gobbi et al., 2000).

Evidence also suggests that regulation of hypertonic saline intake by rats may not be limited to inhibitory effects of serotonin receptor activation in the LPBN (see Table 1). Antagonism of corticotropin releasing hormone in the LPBN significantly enhanced the intake of hypertonic saline suggesting that endogenous corticotropin releasing hormone also acts in the LPBN to inhibit sodium appetite in states of sodium depletion (De Castro e Silva et al., 2006). A robust intake of hypertonic saline solution in response to treatment with furosemide combined with captopril was demonstrated following activation of α_2_-adrenergic receptors and GABA_A_ receptors in the LPBN (Andrade et al., 2004; Callera et al., 2005; Gasparini et al., 2009). More recently, fluid replete rats with bilateral injections of muscimol into the LPBN ingested hypertonic saline which was reduced in response to ICV atropine and atropine or losartan injected directly into the subfornical organ (Asnar et al., 2013; Roncari et al., 2014). Earlier investigations also indicated that cholecystokinin in the LPBN modulated hypertonic saline and water intake in response to an experimentally induced hypovolemic hypotensive states (Menani and Johnson, 1998; Fratucci De Gobbi et al., 2001). Thus, numerous neurotransmitters in the LPBN may interact in the control of sodium appetite.

## Concluding remarks

This review highlights the mounting support for the influential role of the LPBN in regulating the ingestion of both water and hypertonic saline solution. Neurons located within the LPBN provide an inhibitory influence on water intake in response to a range of physiological conditions, with a primary role in responses to challenges associated with increased levels of circulating AngII. Serotonergic mechanisms located within the LPBN have been identified playing a major role in inhibiting sodium appetite and again mostly in response to changes in the renin angiotensin system. However, more recent evidence suggests roles for neurotransmitters in addition to serotonin, including GABA and corticotrophin releasing hormone. Moreover, both neuroanatomical and functional studies identify differential roles for LPB subnuclei in mediating cardiovascular function. Evidence suggests that its integrative function is either mediated by direct baroreflex feedback and or by relaying signals arising from more rostral brain regions. Importantly, it is well known that the critical interaction between sodium levels and water retention can lead to an imbalance and result in higher BP. Given the broad functional significance of the LPBN in modulating fluid intake and cardiovascular function, further examination of this interaction may be key to providing a new understanding of hypertension.

### Conflict of interest statement

The author declares that the research was conducted in the absence of any commercial or financial relationships that could be construed as a potential conflict of interest.
